# PPAR-α Hypermethylation in the Hippocampus of Mice Exposed to Social Isolation Stress Is Associated with Enhanced Neuroinflammation and Aggressive Behavior

**DOI:** 10.3390/ijms221910678

**Published:** 2021-10-01

**Authors:** Francesco Matrisciano, Graziano Pinna

**Affiliations:** Department of Psychiatry, The Psychiatric Institute, College of Medicine, University of Illinois Chicago (UIC), Chicago, IL 60612, USA; matrisci@uic.edu

**Keywords:** social isolation stress, PPAR-α, epigenetics, neuroinflammation, neurosteroids, PTSD, suicide

## Abstract

Social behavioral changes, including social isolation or loneliness, increase the risk for stress-related disorders, such as major depressive disorder, posttraumatic stress disorder (PTSD), and suicide, which share a strong neuroinflammatory etiopathogenetic component. The peroxisome-proliferator activated receptor (PPAR)-α, a newly discovered target involved in emotional behavior regulation, is a ligand-activated nuclear receptor and a transcription factor that, following stimulation by endogenous or synthetic ligands, may induce neuroprotective effects by modulating neuroinflammation, and improve anxiety and depression-like behaviors by enhancing neurosteroid biosynthesis. How stress affects epigenetic mechanisms with downstream effects on inflammation and emotional behavior remains poorly understood. We studied the effects of 4-week social isolation, using a mouse model of PTSD/suicide-like behavior, on hippocampal PPAR-α epigenetic modification. Decreased PPAR-α expression in the hippocampus of socially isolated mice was associated with increased levels of methylated cytosines of PPAR-α gene CpG-rich fragments and deficient neurosteroid biosynthesis. This effect was associated with increased histone deacetylases (HDAC)1, methyl-cytosine binding protein (MeCP)2 and decreased ten-eleven translocator (TET)2 expression, which favor hypermethylation. These alterations were associated with increased TLR-4 and pro-inflammatory markers (e.g., TNF-α,), mediated by NF-κB signaling in the hippocampus of aggressive mice. This study contributes the first evidence of stress-induced brain PPAR-α epigenetic regulation. Social isolation stress may constitute a risk factor for inflammatory-based psychiatric disorders associated with neurosteroid deficits, and targeting epigenetic marks linked to PPAR-α downregulation may offer a valid therapeutic approach.

## 1. Introduction

The COVID-19 pandemic is currently challenging the mental health of the world’s general population, demonstrated by the skyrocketing prevalence of psychological distress and the high incidence of psychiatric disorders [1]. Furthermore, individuals with pre-existing mental disorders, including anxiety, major depressive disorder (MDD), and posttraumatic stress disorder (PTSD), are at higher risk for symptom exacerbation. The emergence of suicidal thoughts and attempts, substance use disorder and addictions, and impulse control disorders is also alarming [2,3,4].

A combination of individual factors, including personal fear of contagion, history of trauma and mental disorders, and environmental factors including financial losses and an obligated social withdrawal without adequate individual-based psychological and economic support or therapeutic strategies may enhance stress-related affective instability in vulnerable populations [5,6,7]. The ability to overcome stressful events or trauma without significant psychophysiological alterations is based on the combination of genetic and epigenetic factors, which are crucial in stress vulnerability [8]. Chronic stress may impair the ability to develop resilience by altering neurobiological factors, including the HPA axis; neurotransmitter systems and functions; and neurosteroid biosynthesis [9,10]. Immune dysregulation and neuroinflammation, evidenced by elevated levels of proinflammatory markers and supported by the finding that anti-inflammatory agents elicit antidepressant effects, are also often presented in psychiatric subjects [11,12]. Identifying the neurobiological factors underlying vulnerability to stress might allow for preventive measures for subjects at risk, and enhance biomarker discovery and novel therapeutic strategies.

Stress affects epigenetic mechanisms that have profound effects on gene expression, including DNA methylation/demethylation and histone acetylation/deacetylation dynamics [13]. DNA methylation is a complex mechanism promoting gene silencing catalyzed by specific “writers”, such as DNA methyltransferases (DNMTs), and highly regulated by the interaction with several proteins, including methyl-cytosine binding proteins (MeCP2) and histone deacetylases (HDACs), and “erasers” like the ten-eleven translocation methylcytosine dioxygenases (TETs) mediating demethylation. How stress affects epigenetic mechanisms with downstream effects on inflammation and emotional behavior remains poorly understood.

Protracted social isolation is a strong form of stress and constitutes a vulnerability factor to model emotional behavioral dysfunctions, including deficits in fear extinction and fear extinction retention [14], which are reminiscent of PTSD endophenotypes in mice [15]. Rodents exposed to social isolation also show increased aggressive behavior scores measured by a tendency to engage in agonistic forms of behavior against a same-sex intruder. Multiple epidemiologic, clinical, retrospective, prospective, and family studies support the hypothesis that individuals with high levels of reactive aggressiveness are at risk for suicidal behavior [16,17,18]. Indeed, aggressiveness is a recognized phenotype of impulse control disorder and suicidality risk. Thus, the socially isolated (SI) mouse represents a valid model to study emotional behavioral dysfunctions of PTSD/suicide risk behaviors in humans [19,20,21]. Importantly, as in PTSD subjects, SI mice show altered neurosteroid biosynthesis, including levels of allopregnanolone (Allo) and its isomer pregnanolone, two potent, positive, allosteric modulators of GABA action at the GABA_A_ receptor, associated with decreased peroxisome proliferator-activated receptor (PPAR)-α protein expression [15]. PPAR-α, a ligand-activated nuclear receptor and transcription factor, regulates cell development, energy homeostasis, and metabolism [22], as well as several pathophysiological processes including inflammation and oxidative stress via toll-like receptor 4 (TLR4)/NF-κB signaling. PPAR-α activation by the endogenous modulator, *N*-palmitoyl-ethanolamine (PEA) induces Allo biosynthesis and a rapid improvement of PTSD-like behavior in SI mice [15], suggesting that PPAR-α may represent a key molecular player upstream of Allo biosynthesis and emotional behavioral regulation.

Here, we studied the methylation status of the PPAR-α gene as a potential epigenetic factor contributing to its expression suppression in SI mice. The mRNA expression of epigenetic marks, including DNMT (1, 3a, and 3b), HDAC (1 and 2), and TET (1–3), as well as pro-inflammatory mediators and cytokines (TLR4, NF-κB, TNFα, and MCP1), were also investigated relative to aggressive behavior in SI vulnerable/aggressive and SI resilient/non-aggressive mice. We showed that altered DNA methylation status and epigenetic marks underlie the lower hippocampus PPAR-α expression in SI mice, increased pro-inflammatory processes promoted by TLR4 activation, and decreased Allo and pregnanolone levels. Interestingly, these changes occurred exclusively in vulnerable SI mice that showed a high aggressive behavior, but not in SI non-aggressive mice. These findings may contribute neurobiological underpinnings relevant to PTSD neurobiology complicated by suicidality or mechanisms for the resilience thereof.

## 2. Results

### 2.1. Decreased PPAR-α Expression in the Hippocampus of SI Mice Correlates with Aggressive Behavior

**Figure 1:**
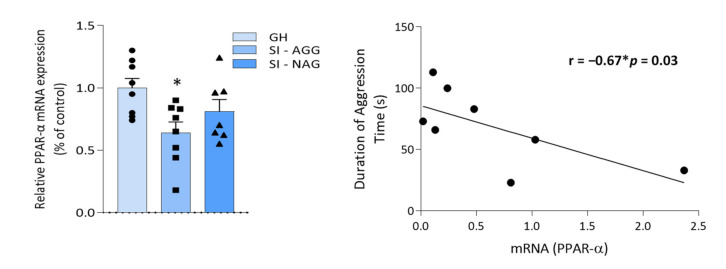
PPAR-α mRNA expression in the hippocampus of socially isolated (SI) mice and its correlation with aggressive behavior. (**A**) A group of mice that failed to develop aggressive behavior (here termed SI non-aggressive, SI-NAG) during protracted social isolation was separated from the SI mice that showed high aggressive behavior scores (SI-AGG). Decreased PPAR-α mRNA expression in the hippocampus of SI-AGG mice was observed when compared with GH or SI-NAG mice (one-way ANOVA: * *p* = 0.03; F = 3.8). Values are means ± S.E.M. of eight control (GH), eight SI-AGG, and seven SI-NAG mice. (**B**) A significant negative correlation exists between PPAR-α mRNA expression and aggressive behavior (Pearson’s *r* = −0.67; * *p* = 0.03, *n* = 8).

We previously showed a decrease in PPAR protein expression in SI mice (15). Here, we selected SI mice that showed high levels of aggressive behavior (indicated as SI-AGG) compared with low-to-non-aggressive (<5 bites/10 min) mice. These SI mice showed resilience to social isolation stress and were termed SI non-aggressive (SI-NAG) mice. We found decreased PPAR-α mRNA levels in the hippocampus of SI-AGG (one-way ANOVA: * *p* = 0.03; F = 3.98; N = 8), whereas no difference was observed in SI-NAG (*n* = 7) compared with GH mice (*n* = 8; Figure 1A). Moreover, the Pearson correlation analysis showed a significant negative correlation between aggressive behavior and PPAR-α mRNA expression in SI-AGG mice (Pearson r = −0.65; * *p* = 0.04; *n* = 8; Figure 1B).

### 2.2. Social Isolation Stress Induces DNA Methylation of PPAR-α CpG-Rich Promoter Regions

**Figure 2:**
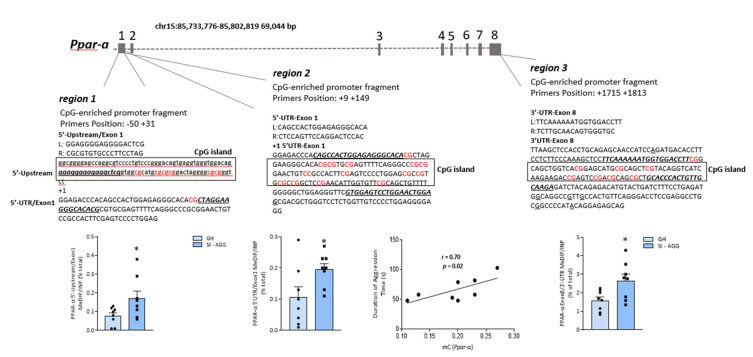
A schematic representation of the mouse *Ppar-α* gene organization. The mouse *Ppar-α* gene is located on chromosome 15 and contains eight exons. Based on the characterization of the human *Ppar-α* gene reported by Pineda Torra (2002) [23], we selected three different fragments of the gene rich in CG dinucleotides within the proximal promoter, 5′-UTR (within exon 1), or 3′UTR portions of the gene and amplified by specific primers using the qPCR technique. The selected region (region 1) consists of a CpG-enriched promoter fragment positioned at −50/+31 bp linking the 5′-upstream promoter region to the 5′-UTR fragment of Exon 1. Region 2 amplified the 5′-UTR and part of exon 1 after the transcription starting nucleotide (+1) positioned at +9 +149 bp, whereas region 3 covered the 3′-UTR and part of the exon 8 positioned at +1715 +1813 bp. The increase in cytosine methylation levels was measured by methylated DNA immunoprecipitation (MeDIP) of the *Ppar-α* gene fragments (region 1: unpaired *t* test: * *p* = 0.04, *t* = 2.23, df = 14, *n* = 8; region 2: * *p* = 0.03; *t* = 2.30; df = 14; *n* = 8; region 3: * *p* = 0.02; *t* = 2.59; df = 14; *n* = 8). In addition, a positive correlation between methylated cytosines (mC) levels of region 2 and aggressive behavior was observed (Pearson’s *r* = 0.70; * *p* = 0.02; *n* = 8).

To test whether a lower PPAR-α mRNA expression was the result of epigenetic changes in the *Ppar-a* CpG-rich fragments, we measured the immunoprecipitated methyl-cytosines of the three regions of the gene analyzed in the hippocampus of the same group of mice. A schematic representation of the *Ppar-a* gene structure and the regions amplified is shown in Figure 2. We found a significant increase in precipitated methylated cytosine (mC) levels in all the three *Ppar-a* gene regions amplified in the hippocampus of SI-AGG mice compared with the control (GH) mice (Figure 2) (region 1: unpaired *t* test two-tailed: * *p* = 0.04; *t* = 2.23; region 2: * *p* = 0.03, *t* = 2.30; region 3: * *p* = 0.02, *t* = 2.59; *n* = 8). In addition, a positive correlation was observed between aggressive behavior and methylation status of the cytosines studied in the upstream region (region 2) of the *Ppar*-α gene (r = 0.70; * *p* = 0.02; *n* = 8; Figure 2). Methylation analysis in SI-NAG mice was not performed because no changes were found in the PPAR-α mRNA expression in these mice.

### 2.3. Altered Expression of Chromatin Remodeling Marks in the Hippocampus of SI Mice

**Figure 3:**
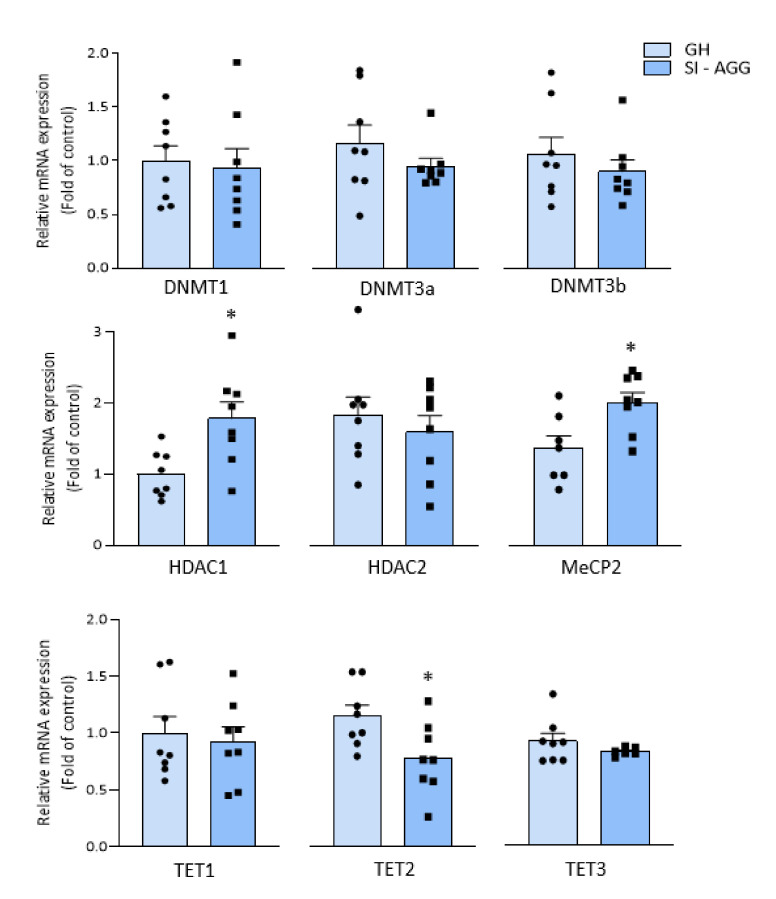
mRNA expressions of DNMTs, HDACs, MeCP2, and TETs in the hippocampus of SI-AGG and GH mice. Increased mRNA of HDAC1 and MeCP2 (Unpaired *t* test two-tailed: HDAC1: * *p* = 0.01; *t* = 2.96; df = 14; *n* = 8; MeCP2: * *p* = 0.01; *t* = 2.81; df = 13; *n* = 7–8) and decreased expression of TET2 (* *p* = 0.02; *t* = 2.46; df = 14; *n* = 8) were found in the hippocampus of SI-AGG mice, whereas no changes were detected in the mRNA of the DNMT enzymes. This evidence corroborates our finding supporting stress-induced epigenetic alterations leading to a PPAR-α gene hypermethylation and a decreased expression in the hippocampus of SI-AGG mice.

We measured the expression of the specific enzymes involved in chromatin remodeling and DNA methylation, such as DNA methyltransferases (DNMT1, 3a, and 3b), HDAC1, and MeCP2, which are involved in DNA methylation processes, and TETs (TE1–3), which play a role as methyl-cytosine dioxygenase in DNA demethylation mechanisms [24], as a result of stress-induced epigenetic alterations. Both the HDAC1 and MeCP2 mRNA levels were higher in the hippocampus of the SI-AGG mice than in the hippocampus of the GH mice (Figure 3; unpaired *t* test two-tailed: HDAC1: * *p* = 0.01; *t* = 2.96; df = 14; *n* = 8; MeCP2: * *p* = 0.01; *t* = 2.81; df = 13; *n* = 7–8). In addition, a decreased mRNA expression of TET2 (* *p* = 0.02; *t* = 2.46; df = 14; *n* = 8) was found, suggesting an imbalance between methylation/demethylation processes. Together, these findings suggest that social isolation leads to changes in epigenetic marks that trigger hypermethylation of the *Ppar-α* gene. No significant changes in mRNA levels of DNMT1, 3, or 3b in the hippocampus of SI-AGG mice were found (DNMT1 GH = 1 ± 1.14; SI-AGG = 0.93 ± 0.18, *p* = 0.78; DNMT3a GH = 1 ± 0.17; SI-AGG = 0.72 ± 0.02, *p* = 0.15; DNMT3b GH = 0.82 ± 0.11; SI-AGG = 0.66 ± 0.04, *p* = 0.23).

### 2.4. Increased Expression of Pro-Inflammatory Markers in the Hippocampus of SI Mice Is Associated with Aggression

To explore whether stress-induced *Ppar-α* gene suppression leads to the activation of neuroinflammatory processes, we measured the mRNA expression of TLR4, NF-κB, TNFα, and MCP-1, which are involved in the PPAR-α anti-inflammatory pathway mediated by NF-κB activation.

**Figure 4:**
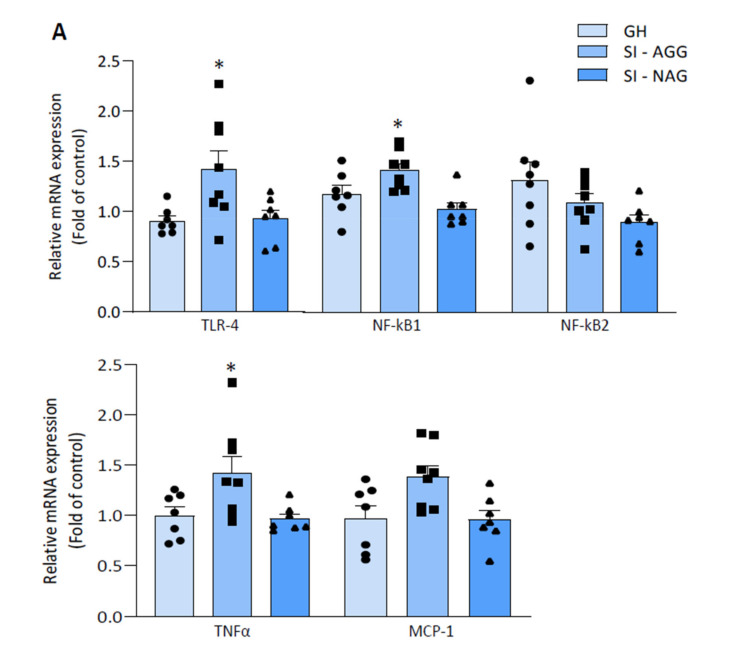

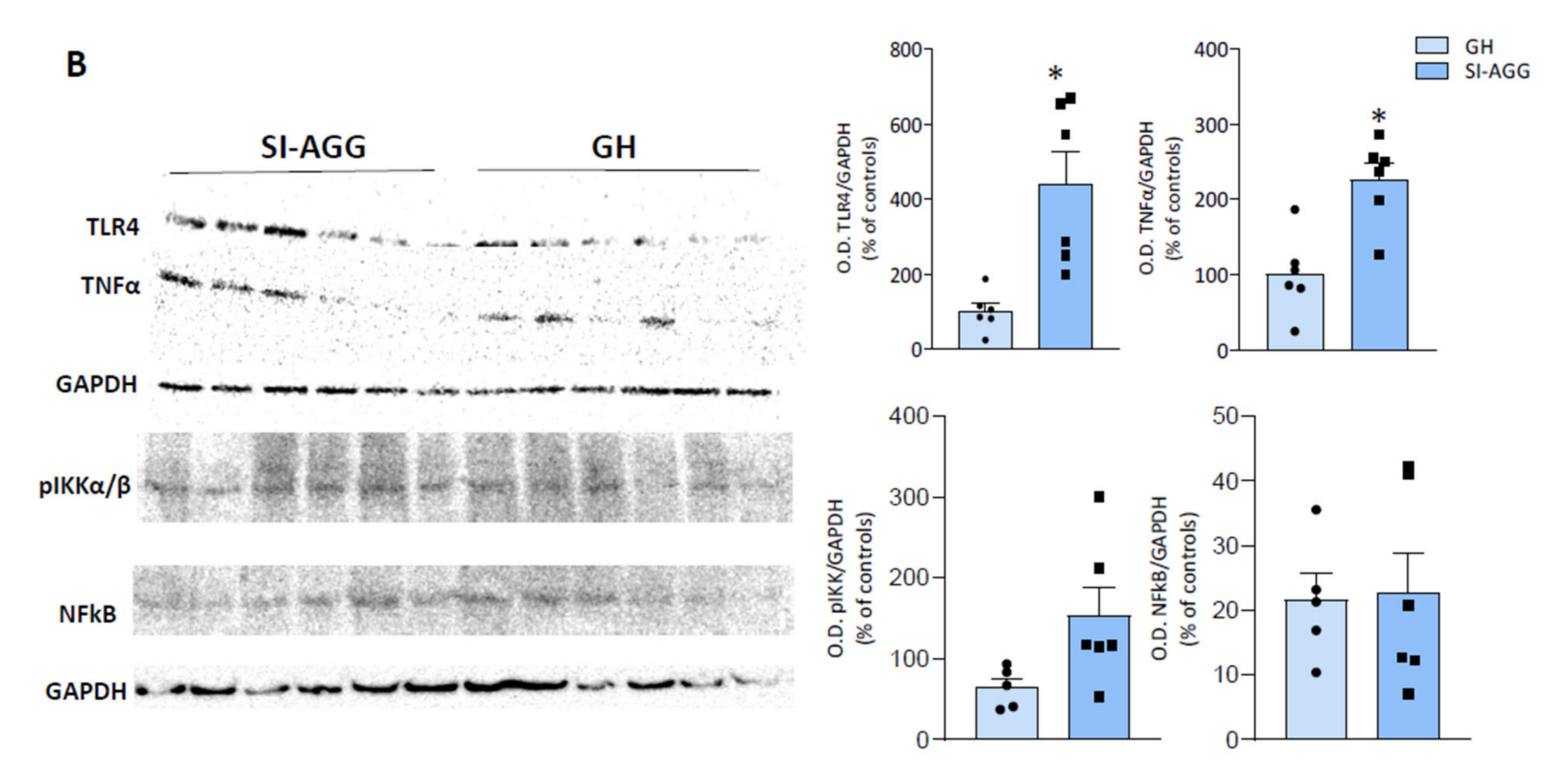
(**A**) TLR4, NF-κB (1 and 2), TNFα, and MCP-1 mRNA expression in the hippocampus of SI-AGG and GH mice. Increased levels of TLR4, NF-κB1, TNFα, and MCP1 observed in SI-AGG mice compared with GH or SI-NAG mice (one-way ANOVA: TLR4: * *p* = 0.03; F = 4.1; TNFα: * *p* = 0.01; F = 5; MCP-1: * *p* = 0.01, F = 4.9; NF-κB1: * *p* = 0.005; F = 2; *n*= 7–8). No changes in NF-κB2 were found. (**B**) Representative immunoblot for TLR4, TNFα, pIKKα/β, and NF-κB protein expression. The optical density (OD) of the bands was quantified and normalized relative to the housekeeping protein GAPDH. The bar graphs were obtained using values as means ± S.E.M. of 5–6 samples for the controls (GH) and 6 samples for SI-AGG. The samples were loaded in the same immunoblot, and the values were calculated relative to the samples from GH mice. Individual values are shown in the relative graph plots. For the significance, values were analyzed using unpaired two-tailed *t* test.

In the hippocampus of SI-AGG mice, the TLR4, NF-κB1, TNF-α, and MCP-1 mRNA levels were increased (one-way ANOVA: TLR4: * *p* = 0.03; F = 4.1; NF-κB: * *p* = 0.005; F = 2; TNFα: * *p* = 0.01; F = 5; MCP-1: * *p* = 0.01, F = 4.9; *n* = 7–8), which supports our hypothesis of genomic effects on the stimulation of neuroinflammatory processes associated with a PPAR epigenetic repression. Of note, the mRNA expression of TLR4, TNFα, MCP-1, and NF-κB1 were lower in SI-NAG mice compared with GH mice (Figure 4A). We also measured the protein expression of TLR4, TNFα, pIKKα/β, and NF-κB in the hippocampus of SI-AGG mice. An increase in the TLR4, TNFα, and pIKKα/β protein expression was found in SI-AGG mice compared with GH mice (unpaired *t* test two-tailed: TLR4 = * *p* = 0.006, *t* = 3.24; TNFα = * *p* = 0.006, *t* = 3.56; pIKKα/β = * *p* = 0.03, *t* = 2.13; *n* = 5–8) whereas no differences were detected for the NF-κB protein expression (unpaired *t* test: *p* = 0.88, *t* = 0.149) (Figure 4B).

### 2.5. Neurosteroid Levels in Aggressive and Non-Aggressive SI Mice

**Figure 5:**
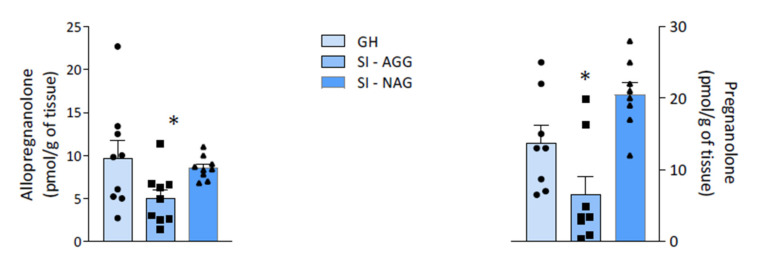
The levels of allopregnanolone (Allo) and pregnanolone (PA) in the hippocampus of SI and GH mice. A significant decrease in both Allo and PA levels was found in the hippocampus of SI-AGG mice (unpaired *t* test: Allo = * *p* = 0.04, *t* = 2.18, df = 16; PA = * *p* = 0.04, *t* = 1.78, df = 12).

Decreased Allo and pregnanolone levels were specifically observed in the hippocampus of SI-AGG mice (unpaired *t* test: Allo = * *p* = 0.04, *t* = 2.18; PA = * *p* = 0.04, *t* = 1.78; Figure 5).

## 3. Discussion

This study provides the first evidence of a PPAR-α epigenetic regulation induced by DNA methylation in the mouse brain. In the hippocampus of SI-AGG mice, our results show altered epigenetic marks, including (i) increased levels of methylated cytosines of the *Ppar-α* gene (Figure 2); (ii) increased expression of HDAC1 and MeCP2, which belongs to the methyl-CpG-binding domain protein family (Figure 3); and (iii) a reduced TET2 expression. Our main finding of the hypermethylated *Ppar-α* gene and its expression down-regulation is thus supported by the increase in HDAC1 and MeCP2 and decrease in TET mRNA expression, which favor hypermethylation of the CG fragments within the promoter regions of the *Ppar-α* gene. This evidence strongly supports a genomic effect induced by isolation stress and the involvement of DNA methylation mechanisms. Significantly decreased PPAR-α mRNA expression, downregulated brain Allo and pregnanolone levels, increased pro-inflammatory factors (NF-κB and TLR-4), and increased protein expression of the pIKKα/β and TNFα were associated with vulnerable SI mice expressing high aggression scores. These data support a *Ppar-α* gene hypermethylation as a leading mechanism to decreased hippocampus PPAR-α expression and favoring neuroinflammation mediated by the NF-κB/TLR-4 pathway. This evidence corroborates findings that deficits in PPAR-α act by promoting proinflammatory processes and dysfunctional behaviors exacerbated by stress exposure, including social isolation.

PPAR-α is deeply involved in several physiological and pathological conditions, including regulation of the mitochondrial and proteasomal function, neuroinflammation, oxidative stress, and neurodegeneration, which are considered key pathogenetic mechanisms involved in neurodegenerative diseases and stress-related disorders, including anxiety, MDD, PTSD, postpartum depression, and suicide [25,26,27]. PPAR-α is also known to exhibit anti-inflammatory effects and a neuroprotective activity by modulating the expression of inflammatory mediators, including IκB, an NF-κB inhibitor, leading to the suppression of cytokine production [28]. NF-κB resides inactively in the cytoplasm associated with inhibitory IκB proteins. Upon NF-κB pathway activation, the IκB protein is degraded and the NF-κB complex moves into the nucleus and modulates the expression of the target genes. IκB degradation is mediated by the IκB kinase (IKK) complex, which, via phosphorylation, induces IκB proteasomal degradation [29]. In our results, the pIKK protein expression in increased in SI aggressive mice, as shown in Figure 4, supporting the hypothesis of NF-κB activation.

### 3.1. Epigenetic Mechanisms Affecting PPAR-α Expression during Social Isolation

The mouse *Ppar-α* gene, located on chromosome 15, comprises a total of eight exons, with a 5′-untranslated region (5′UTR) encoded by exons 1 and 2, and part of exon 3, whereas the remaining part of exon 3 and exons 4–8 contribute to the coding region. The last portion of exon 8 (232 bp) contributes to the 3′untraslated region (3′UTR) [30]. Both 5′UTR and 3′UTR constitute regulatory regions for gene transcription [23]. These regions are also characterized by a high CpG density detected by MethPrimer. The altered expression of PPAR-α, which is involved in the recruitment of epigenetic factors (class III NAD^+^-dependent HDAC Sirt1) [31], may affect the expression of the target genes, including genes encoding for neurosteroidogenic enzymes and pro-inflammatory markers.

Despite the numerous implications of PPAR-α in physiological and pathological conditions, little is known about its epigenetic regulation, especially within the brain. Few studies have investigated the epigenetic regulation of the *Ppar-a* gene promoter in obesity, cardiac and intestine pathologies, and liver disease, where the DNA methylation of PPAR-α has been suggested as a therapeutic approach for non-alcoholic fatty liver disease [32]. Thus, this study contributes the first demonstration that PPAR-α is susceptible to stress-induced epigenetic regulation in the brain.

Despite the fact that there was no difference in the DNMT mRNA expression in SI-AGG mice, the increased expression of MeCP2 and HDAC1, and reduced TET2, which together promote DNA methylation [31], suggest an imbalance in the dynamics between DNA methylation/demethylation in the hippocampus of SI-AGG mice. HDAC inhibitors are intensively investigated for specific clinical applications including cancer, neurodegenerative disorders, and as cognitive enhancers [31,33,34,35,36]. Tet genes are decreased in chronic inflammatory conditions and animal models thereof, causing aberrant DNA methylation [37]. Reduced TET2 expression has also been implicated in altered neurogenesis and cognitive impairment [38,39]. These findings might favor pharmacological interventions aimed at inhibiting DNA methylation targeting the PPAR-α gene as a novel approach for stress-related neuroimmune disorders, which include neuropsychiatric disorders. Administering HDAC inhibitors may represent a valuable pharmacological approach to modulate PPAR-α expression and reinstate its functional role as an anti-inflammatory and behavioral regulator [40,41].

### 3.2. PPAR-α/Neurosteroid Downregulation and Increase in Pro-inflammatory Mediators in SI Mice

Preclinical and clinical studies show decreased Allo and pregnanolone levels in depression and PTSD, while the enhancement of their concentrations is associated with symptom improvement [42,43,44]. Of note, IV Allo (i.e., brexanolone) is FDA approved to treat post-partum depression after pivotal Phase 3 clinical trials, demonstrating high efficacy and safety profiles [45]. In SI mice, decreased Allo levels are inversely correlated with anxiety-like behavior, exaggerated contextual fear responses, and aggression, which is the most prominent behavioral trait to model suicide risk in humans [15,19]. Allo binds primarily at extrasynaptic GABA_A_ receptors in corticolimbic pyramidal neurons [46,47], which is regarded as the mechanism underlying the improvement of behavioral dysfunction in human and animal studies [48,49]. These behavioral effects are also reproduced by administration with the PPAR-α endogenous modulator, PEA, and the synthetic ligand, fenofibrate, in SI mice, which upregulate neurosteroidogenic enzymes and proteins and Allo levels in corticolimbic areas [15], including the hippocampus and the amygdala, with well-established roles in emotional behavior regulation. Downregulation of Allo and its equipotent GABAergic isomer, pregnanolone, occurs specifically in SI mice that express aggressive behavior, but not in “resilient” mice that fail to express behavioral impairment. In the hippocampus of SI-AGG mice, the expression of TLR-4 and NF-κB, and of cytokines TNF-α and MCP1 increased, signifying a pro-inflammatory condition. Previous studies have provided evidence that an increased TLR-4 expression underlies a downstream activation of the NF-κB signaling cascade, promoting pro-inflammatory cytokines [50].

TLRs are immune receptors expressed in innate immune cells (dendritic cells and macrophages) and in non-immune cells (fibroblasts and epithelial cells) that are involved in proinflammatory responses and neuroinflammation [51]. In neurons, microglia, and astrocytes, Allo mediates potent anti-inflammatory effects through NF-κB signaling inhibition [52]. Allo and its precursor, pregnenolone, inhibit TLR-4 signaling and the production of pro-inflammatory mediators by a mechanism independent of Allo activation of the GABA_A_ receptor [53,54], suggesting a direct link between neurosteroidogenesis and TLR4-mediated inflammation. Activation of the TLR4/MyD88 signaling pathway induces the expression of pro-inflammatory cytokines, whereas Allo prevented this effect by inhibiting TLR-4 binding to the MyD88 adaptor and to its specific endogenous ligand, lipopolysaccharide (LPS) [53]. Allo may also enhance LPS degradation [54]. Thus, decreased hippocampus Allo levels in SI mice may play a role in the following: (1) the inflammatory processes resulting from PPAR-α downregulation and enhancement of NF-κB signaling, and (2) the behavioral dysfunction arising from the downregulation of Allo-modulated GABAergic neurotransmission. The mechanisms of stress-induced Allo biosynthesis downregulation in the contest of impaired NF-κB and TLR-4 pathways remain to be clarified, as well as whether TLR-4 abnormal activation occurring in stress-induced models of affective disorders [55] is linked to the downregulation of brain Allo levels. Indeed, exposure to TLRs agonists suppresses PPAR-α expression via a NF-κB-dependent signaling in astrocytes [56], raising the question of whether this can be an additional mechanism responsible for low Allo levels in pathophysiological conditions characterized by elevated inflammation and affective symptoms.

### 3.3. PPAR-α/TLR4/Allo Pathway as a Potential Biomarker and Pharmacological Target in Stress-Induced Behavior

We previously reported that SI mice express aggression and altered fear responses associated with decreased corticolimbic Allo levels and lower PPAR-α protein content [15]. This is associated with decreased levels of the Allo biosynthetic enzyme, 5α-reductase type I (5α-RI) [57]. This study shows that social isolation also decreases pregnanolone levels specifically in vulnerable SI AGG mice. Given the role PPAR-α plays in neurosteroidogenesis [15,58], our study suggests that stress-induced epigenetic suppression of PPAR-α may underlay both the enhancement of proinflammatory markers, mediated by TLR4-activation, and neurosteroid biosynthesis downregulation. Whether altered PPAR-α expression directly interferes with the recruitment of epigenetic factors [59] involved in the regulation of Allo biosynthetic genes remains to be further analyzed. A significant correlation was found between mRNA PPAR-α-suppression, its methylation status, and aggression, as well as between the levels of neurosteroids and the expression of aggression. As such, our results suggest that an altered PPAR-α/TLR4/Allo pathway may offer biomarkers of mood instability and stress-induced resiliency deficits. Consistently, PPAR-α hypermethylation induced by protracted stress may provide a new neurobiological factor underlying stress-induced behavioral abnormalities in vulnerable populations. Epigenetic changes are linked to maladaptive stress responses and psychiatric disorders [60]. Hypermethylation of the glucocorticoid receptor (GR) gene (*NR3C1*) promoter was reported in the hippocampus of suicide victims with a history of childhood abuse compared with those without early-life trauma and with control subjects [61]. Increased methylation of the alpha-1 subunit of the GABA_A_ receptor gene (*Gabra1*) promoter was also found in the prefrontal cortex of suicide victims [62]. Pharmacoepigenetic strategies aimed to increase PPAR-α expression may be useful in populations that show hypermethylation and suppression of PPAR-α caused by chronic stress conditions. It remains to be investigated if the PPAR-α hypermethylation status can be revealed in the peripheral blood mononuclear cells (PBMC) of stressed animals and subjects with psychiatric conditions. The goal to connect psychiatric symptoms with biological alterations as an objective indicator of pathology is still unmet. The discovery of blood or saliva biomarkers will represent a breakthrough in the management of PTSD and suicide, as well as in psychiatric disorders in general. Several biomarker candidates, including BDNF and HPA-axis alterations with increased levels of corticotropin-releasing hormone (CRH) and cortisol, have been proposed, but none have yet been established for clinical screening [63]. A low expression of PPAR-α and an increased expression of TLR-4 and pro-inflammatory cytokines have been reported in peripheral pathological conditions and metabolic inflammation [63,64]. Thus, establishing a correlation between the peripheral and central expression and DNA methylation status of PPAR-α/TLR-4/Allo in animal models and clinical studies should be prioritized.

PEA is a lipid mediator that exerts anti-inflammatory effects by direct activation of PPAR-α as a main molecular target [65]. The PEA stimulation of PPAR-α induces corticolimbic Allo biosynthesis, an effect that is directly associated with the improvement of PTSD/suicide-like phenotypes in SI mice [15,66]. The administration of PEA also stimulates PPAR-α protein and 5α-RI expression [15,67]. Although the underlying mechanisms are not yet fully understood, these findings suggest a possibility that PEA may reverse stress-induced epigenetic modifications, leading to an up-regulation of PPAR-α expression. While these mechanisms remain to be studied, treatment with PEA to alleviate neuroinflammatory-based neuropsychiatric disorders could provide a reasonable therapeutic strategy. Importantly, PPAR-α has been recently investigated as a target for natural and synthetic compounds that exert an anti-inflammatory and neuroprotective effect in rodent models for neurodegenerative diseases [26,68]. Ongoing clinical trials are studying the efficacy of natural micronutrients, such as genistein or synthetic PPAR-α ligands for the treatment of neuropsychiatric disorders, including Alzheimer’s disease, bipolar depression, and alcoholism [26,69,70].

## 4. Methods and Materials

### 4.1. Animals

Male Swiss-Webster mice (Charles River Laboratories; 21–23 days old at arrival) were maintained under a 12-h dark/light cycle (light on at 06:00 a.m. and off at 18:00 p.m.) and were provided food and water ad libitum. SI mice were housed individually in a cage (24 × 17 × 12 cm) for 4–6 weeks, while group-housed (GH) mice were housed in groups of 5. The vivarium temperature was 24 °C and the humidity 65%. Depending on the study, the number of mice for each experimental group varied from 6 to 12 (see figure legends). Experimental protocols were approved by the Office and Animal Care and Institutional Biosafety Committee and the Office of the Vice Chancellor for Research of the University of Illinois at Chicago (ACC Number: 18-241, approved on 15 February 2019).

### 4.2. Behavioral Analysis

Aggressive behavior was analyzed using the resident-intruder test, as previously described [20]. Briefly, a naïve, sex and weight-matched “intruder” mouse was introduced into the “home cage” of the experimental “resident” mouse, which was housed in social isolation for 4 weeks, and resident–intruder interactions were videotaped for 10 min. Several behaviors were considered, such as number of attacks, bites, wrestling, and duration of attacks, as an index of aggressive behavior. The results were expressed as the total duration of aggression during the 10 min observation period.

### 4.3. Brain Neurosteroid Measurements

Extraction, derivatization, and quantification of Allo and pregnanolone were described previously [71]. After the addition of deuterium-labeled internal standards to tissue samples, steroids were extracted, purified, and separated by high-pressure liquid chromatography (HPLC, Spectra-Physics DSP8800, Milpitas, CA, USA). After derivatization with HFBA, gas chromatography–mass spectrometry (GC−MS 7820A/5977, Agilent Technologies, Santa Clara, CA, USA) analysis in the standard electron impact mode was performed [20].

### 4.4. Real-Time Polymerase Chain Reaction (RT-PCR)

Total mRNA was extracted from mouse hippocampus using the miRNeasy kit (Qiagen, Valencia, CA, USA). The extracts were treated with DNAse and RT-PCR was performed as previously described [26] using Applied Biosystems RT-PCR System (AriaMx: Agilent Technologies, Santa Clara, CA, USA) with SYBR Green master mix (Brilliant III Ultra-Fast: Agilent Technologies). Primer sequences used for the amplification of targeted cDNA are listed in Table 1. The relative mRNA expression values were calculated as the fold of controls using β-Actin as the internal control for sample normalization.

### 4.5. Western Blotting

Western blotting was performed as previously described [15]. Homogenized tissue in a RIPA lysis buffer containing protease and phosphatase inhibitors (Millipore Sigma) was centrifuged and the supernatant was used for protein determination. First, 100 µg of proteins were diluted in Laemmli buffer, run on 4–15% Bis-Tris gel (Bio-Rad Laboratories, Inc, Hercules, CA, USA) and transferred to polyvinylidene difluoride membranes (Bio-Rad Laboratories, Inc). After blocking with 5% dry milk for 1 h, the membranes were incubated overnight at 4 °C with an anti-TLR4 antibody (diluted 1:1000 with 5% BSA in TBST, cat no. 14358S; Cell Signaling Technology, Inc. Danvers, MA, USA), anti-TNFα antibody (diluted 1:500 with 5% BSA in TBST, cat no. 11948S; Cell Signaling Technology, Inc.), anti-NF-κB p65 antibody (diluted 1:500 with 5% BSA in TBST, cat no. 8242; Cell Signaling Technology, Inc.), or anti-P-IKK-alpha (S176)/IKK-beta (S177) antibody (diluted 1:1000 with 5% BSA in TBST, cat no. 2078; Cell Signaling Technology, Inc.), and were further processed with anti-rabbit secondary antibodies (1:2000 in 5% dry milk for 1 h at RT; cat no. 7074; Cell Signaling Technology). Immunocomplexes were revealed by enhanced chemiluminescence with an Immobilon Western Chemiluminescent HP Substrate Kit (ECL Plus (PS-3), Lumigen) using a LI-COR (Lincoln, NE) ODISSEY System, and were analyzed by Image Studio 5.2 (iS5.2) software (LI-COR). In all blots, the optical density of the TLR4, TNFα, NF-κB, and P-IKK bands were quantified and normalized relative to the housekeeping GAPDH (Cell Signaling Technology; diluted 1:1000 with 5% dry milk in TBST, overnight at 4 °C).

### 4.6. PPAR-α Gene Methylation Analysis

DNA methylation levels of the *Ppar-α* gene were analyzed by methyl-DNA immunoprecipitation (MeDIP) using the MagMeDIP kit (Diagenode, Denville, NJ) after genomic DNA extraction of the mouse hippocampus (QIAmp DNA Micro Kit, Qiagen, Germantown, MD, USA), as previously described [72,73]. The *Ppar*-α gene was retrieved at the Ensemble website (http://genome.ucsc.edu/ accessed on 25 August 2020), and primers were designed (gene symbol: *PPARA*) using genome.ucsc.edu (NSMUST00000109422.7 chr15: 85734975-85735143). Three different regions of the *Ppar-α* gene CpG-enriched sites were analyzed: (i) region 1: 5′-upstream/Exon1; (ii) region 2: 5′-UTR/Exon1; and (iii) region 3: 3′-UTR/Exon8. The specific DNA sequence was amplified by qPCR analysis using the following primers: region 1: F: GGAGGGGAGGGGACTCG; R: CGCGTGTGCCCTTCCTAG; region 2: F: CAGCCACTGGAGAGGGCACA, R: CTCCAGTTCCAGGACTCCAC; region 3: L: TTCAAAAAATGGTGGACCTT, R: TCTTGCAACAGTGGGTGC. A representative the *Ppar-α* gene structure is schematized in Figure 2. The percent methylated vs. unmethylated promoter was calculated using the following equation: % (meDNA-IP/total input) = 2^[(Ct(10%input) – 3.32) – Ct (meDNA – IP)] × 100% (MagMeDIP kit instruction manual, Diagenode), and expressed as the percent of total (INP).

### 4.7. Statistical Analysis

The results were analyzed using a two-tailed unpaired *t*-test for the results in Figure 2, Figure 3, and Figure 4B; one-way ANOVA followed by Tukey’s multiple comparison test for the results in Figure 1, Figure 4A, and Figure 5B; and two-tailed Pearson’s correlation test for the results in Figure 1B and Figure 2. The F-value was determined by ANOVA using GraphPad Prism 8.2.1. Values are expressed as the means ± SEM of 6–8 mice for the biochemical analysis and 8–10 mice for the behavioral tests. Significance was set at * *p* < 0.05. The correlation results were obtained using two-tailed Pearson’s correlation analysis (for the results in Figure 1B and Figure 2B). For the biochemical analysis, we used eight samples per experimental group (GH and SI-AGG) and seven samples for SI-NAG. The specific number of values for each experiment is indicated in the respective results sections.

## Figures and Tables

**Table 1:** Primer sequences for qPCR analysis.

Gene	Forward	Reverse
mRNA expression		
*β-Actin*	5′-TGTGATGGTGGGAATGGGTCAGAA	5′-TGTGGTGCCAGATCTTCTCCATGT
*Ppar-alpha*	5′-CACAGACACCCTCTCTCCAG	5′-AGCCCTTACAGCCTTCACAT
Epigenetic Marks		
*HDAC1*	5′-CACAGACACCCTCTCTCCAG	5′-AGCCCTTACAGCCTTCACAT
*HDAC2*	5′-GCTTGCCATCCTCGAATTAC	5′-CCCTCAAGTCTCCTGTTCCA
*MeCP2*	5′-GGTTGTCTCCACTGCTACTTAC	5′-GCTAACTTGGGTGCTGATCT
*TET1*	5′-TTGCCCAGACCATAAGGAAC	5′-GTGGTGACACTCATGGCATC
*TET2*	5′-GTTCTCAACGAGCAGGAAGG	5′-TGAGATGCGGTACTCTGCAC
*TET3*	5′-TCCGGATTGAGAAGGTCATC	5′- CCAGGCCAGGATCAAGATAA
*DNMT1*	5′-CCACCACCAAGCTGGTCTAT	5′-TGCCACCAAACTTCACCATA
*DNMT3a* *DNMT3b* Toll-Like Receptors	5′-ACCAGGCCACCTACAACAAG 5′-ACTTGGTGATTGGTGGAAGC	5′-TGCTTGTTCTGCACTTCCAC 5′-CCAGAAGAATGGACGGTTGT
*TLR4*	5′-GGCAGCAGGTGGAATTGTAT	5′-AGGATTCGAGGCTTTTCCAT
Inflammatory marks		
*TNFα*	5′- ACGGCATGGATCTCAAAGAC	5′-GTGGGTGAGGAGCACGTAGT
*MCP-1*	5′-CCCAATGAGTAGGCTGGAGA	5′-TCTGGACCCATTCCTTCTTG
*NFkB1*	5′- CACCTAGCTGCCAAAGAAGG	5′ GCAGGCTATTGCTCATCACA
*NFkB2*	5′- GATCTCCCGAATGGACAAGA	5′ GAACCGAACCTCAATGTCGT
*Ppar-alpha gene*		
**(−50 to +31 bp)**	5′- GGAGGGGAGGGGACTCG	5′- CGCGTGTGCCCTTCCTAG
**(+9 +149 bp)**	5′- CAGCCACTGGAGAGGGCACA	5′- CTCCAGTTCCAGGACTCCAC
**(+1715 +1813 bp)**	5′- TTCAAAAAATGGTGGACCTT	5′- TCTTGCAACAGTGGGTGC

## Data Availability

All data are presented and available in the manuscript. The corresponding author is available to provide any additional information on data presented in this study.
